# SOMM: A New Service Oriented Middleware for Generic Wireless Multimedia Sensor Networks Based on Code Mobility

**DOI:** 10.3390/s111110343

**Published:** 2011-10-31

**Authors:** Mohammad Mehdi Faghih, Mohsen Ebrahimi Moghaddam

**Affiliations:** Electrical and Computer Engineering Department, Shahid Beheshti University, G. C., Tehran 1983963113, Iran; E-Mail: me.faghih@mail.sbu.ac.ir

**Keywords:** middleware, Wireless Multimedia Sensor Networks, service oriented architecture, code mobility, TinyOS

## Abstract

Although much research in the area of Wireless Multimedia Sensor Networks (WMSNs) has been done in recent years, the programming of sensor nodes is still time-consuming and tedious. It requires expertise in low-level programming, mainly because of the use of resource constrained hardware and also the low level API provided by current operating systems. The code of the resulting systems has typically no clear separation between application and system logic. This minimizes the possibility of reusing code and often leads to the necessity of major changes when the underlying platform is changed. In this paper, we present a service oriented middleware named SOMM to support application development for WMSNs. The main goal of SOMM is to enable the development of modifiable and scalable WMSN applications. A network which uses the SOMM is capable of providing multiple services to multiple clients at the same time with the specified Quality of Service (QoS). SOMM uses a virtual machine with the ability to support mobile agents. Services in SOMM are provided by mobile agents and SOMM also provides a t space on each node which agents can use to communicate with each other.

## 1. Introduction

In recent years, the availability of low-cost hardware such as CMOS cameras and microphones that are able to ubiquitously capture multimedia content from the environment has enabled the development of Wireless Multimedia Sensor Networks (WMSNs); networks of wirelessly interconnected devices that allow retrieving video and audio streams, still images, and scalar sensor data [1]. As time has elapsed, WMSNs have become more and more popular and consequently these networks are used in different domains such as multimedia surveillance, health care, traffic avoidance, environmental monitoring, and industrial process control. This wide range of applications has intensified the need for a programming framework that can help programmers overcome the increasing complexities of applications which stem from the distributed nature of applications and also the need for mechanisms to handle harsh operating conditions such as unreliable wireless communications, node failures, and ultra limited available resources. But, the APIs provided by current operating systems available for WSNs are low level and as a result, the development of applications for these networks is a complex, costly, and time consuming task. Due to this dilemma, WMSNs are usually used in an *ad hoc* manner, and the developed applications for these networks usually consist of static parts which are hard to modify and reuse [2]. These characteristics not only reduce WMSN scalability, but also decrease the modifiability of the networks. For example, if there is a need to modify a program, all nodes of network should be reprogrammed and since physical access to nodes may not be easy due to large number of nodes or impassable environments, reprogramming of nodes must be done remotely. Remote reprogramming is a power consuming task for the reason that radio energy consumption is very high and the compiled program (which is relatively large) must be sent to all nodes.

One way to overcome the complexities of application development is using middle-wares, but because of limited resources available in a WSN, it is not possible to use traditional middle-wares in these networks. Therefore, many middle-wares have been proposed for WSNs with the ultimate goal of increasing programming abstraction level and as a result decreasing the development and maintenance cost of WSN programs [2–4]. However, none of them have been specially designed for WMSNs which have some particular characteristics that influence the design of network and as a result the design and implementation of middle-ware. For example, the need for application specific QoS, high bandwidth demand, multimedia source coding techniques, and multimedia in-network processing are some of unique characteristics of WMSNs that the designer of middleware should consider them [1]. Generally, the design and development of a successful middleware for WSN is not trivial. It needs to deal with the many challenges dictated by the characteristics of WSNs on the one hand and the applications on the other hand. Some of challenges in which a middleware designer might face are [5]:

- Managing limited resources
- Scalability and network topology
- Network heterogeneity
- Quality of service
- Security

In this paper, we propose a service oriented middleware named SOMM which specially designed for WMSNs. In the design of the proposed middleware, the mentioned challenges have been considered. The main goal of this middleware is to support multimedia transmission in WMSNs while decreasing the cost of application development and improving network modifiability and scalability. To this end, SOMM taks advantage of virtual machine and code mobility. SOMM structures an application in terms of mobile agents which provide services to each other to accomplish their tasks. SOMM also provides localized tuple spaces as the tools for communication between agents. In addition, some features are provided in SOMM to support QoS requirements of WMSNs. Therefore, it has some advantages with regard to others as follows:

- It provides a highly scalable platform by using SOA and the concepts of code repositories and service registries.
- It increases the energy efficiency in the case of application updating and node reprogramming by using mobile agents and code repositories.
- Modifiability in SOMM is supported via mobile agents and code repositories.
- It is capable of handling heterogeneous nodes with different capabilities and also it makes possible to have different platforms with different operating systems in the network if needed.

The rest of the paper is organized as follows. Section 2 shows some related works. The SOMM design model is described in Section 3. In Section 4, the application programming interface of SOMM middleware has been presented. Section 5 demonstrates the implementation details of SOMM and finally Sections 6 and 7 show an assessment of SOMM design and our concluding remarks, respectively.

## 2. Related Works

There have been various works addressing high-level WSN middle-wares but to the best of our knowledge, there is no WMSN middleware in the literature. The current WSN middle-ware programming approaches can be classified into low-level programming models and high-level programming models [6]. Middle-wares like Mate [7,8], Impala [9] and Agilla [3,10] which use a low-level programming model, take a platform-centric view and focus on abstracting hardware and allowing flexible control of nodes. High-level programming models like TinyDB [4], MiLAN [11], Cougar [12] and Kairos [13] take an application-centric view instead of the platform-centric view and address how easily application logics can be programmed. High-level programming models are further divided into two types: group-level abstraction and network-level abstraction. Group-level abstractions provide a set of programming primitives to handle a group of nodes as a single entity while network-level abstractions, also known as macro-programming, treat the whole network as a single abstract machine.

Mate [7,8] is included in the class of middleware systems that uses a virtual machine for sensor networks. Mate is implemented on top of TinyOS [14] and allows developers to easily change instruction sets, execution events, and virtual machine subsystems. Mate uses codes broken into capsules of 24 byte-long instructions. This benefits large programs, which are made up of multiple capsules and, thus, easily injected into the network using flooding approach. Although this middleware has a concise and simple programming model, its energy consumption is high for long running programs. Agilla [3,10] is based on Mate and extends that approach by providing mechanisms for better injection of a mobile code into the sensor network to deploy user application. Mobile agents can intelligently move or clone themselves into the desired locations based on network changes. This method is more suitable than the flooding mechanisms that Mate uses for the same purpose (issues relevant with the incorporation of mobile agents in WSNs and WMSNs have been thoroughly investigated in research works such as [15–18]). Impala [9] is a middleware designed for the ZebraNet project [19] and its goal is to enable application modularity, adaptability to dynamic environments, and reparability. Its modular design allows easy and efficient on-the-fly reprogramming via wireless channel. However, Impala is designed to run only on pocket PC handhelds and its nature is not suitable for devices with limited resources.

As we mentioned earlier, Cougar [12] and TinyDB [4] fall within the category of high-level abstractions for sensor network programming. They are designed for use by relatively simple data collection applications such as environmental monitoring applications. They allow users to issue queries in a declarative SQL-like language. Both Cougar and TinyDB are concerned with power conservation and providing query processing strategies that aim to conserve resources but, TinyDB is more sophisticated than Cougar [20]. TinyDB can calculate the frequency of sampling that is required to extend the battery life of a node, and also uses a routing structure called a semantic routing tree to help sensor nodes accurately determine when queries need to be routed to their children in the routing tree. Although sensor database systems are easy to use, a key limitation of these systems is the assumption that sensor nodes are largely homogeneous and therefore they are not suitable for heterogeneous sensor networks.

Different research teams have recently developed macro-programming languages and service-oriented approaches for sensor networks. Kairos [13] employs a macro-programming approach to manage sensors by enabling developers to translate global program behavior to local node behavior and provides programming abstractions form anipulating individual nodes, accessing their neighbors, and acquiring their data. Kairos focuses on providing a small set of constructs, containing only abstractions for nodes, one-hop neighbors, and remote data access and it is language independent so that it can be implemented as an extension to existing programming languages.

MiLAN [11] middleware takes a different approach to the previously discussed solutions in that it builds on existing networking and service discovery protocols using a plug-in mechanism to integrate arbitrary protocols. It provides a data service that supports QoS. Applications submit a query and specify their sensing and QoS requirements to the middleware in terms of graphs describing sensor quality of service and state-based variable requirements. In response to a query, MiLAN creates an execution plan, which specifies the source nodes and the routing tree, such that satisfies the QoS requirement while maximizing energy efficiency.

However middle-wares play an essential role in easy development of applications, providing a useful communication protocol may be a challenge in literature. For example PEMuR has been proposed as a dual scheme protocol for efficient video communication in [21]. This protocol aims at both energy saving and high QoS attainment by combining an energy aware hierarchical routing protocol with an intelligent video packet scheduling algorithm. The routing protocol tries to select the most energy efficient paths and manages the network load according to the residual energy of the nodes. Additionally, the packet scheduling algorithm enables the reduction of the video transmission rate with the minimum possible increase of distortion.

TinySOA [22,23] facilitates the use of wireless sensor networks in traditional application development by using a service-oriented model. It provides a set of Web services and tools which make possible to send and receive information from a deployed sensor network. TinySOA architecture consists of four main parts: TinySOA Node, Gateway, Registry, and Server. When one of the sensor network nodes turns on, it detects and identifies what sensor types are available and announces them by sending a registration message to the Gateway. The Gateway uses the gathered sensor types to register this information into the Registry. Once the sensor types are registered, the Gateway subscribes itself to the services provided by the sensor nodes. When sensor nodes receive the subscription message, they start constantly sending sensed data to the Gateway. The Gateway records sensor readings into a subcomponent called Historical Registry. The Server uses the Registry to prepare a Web service. This Web service leads methods to query for sensor readings. When a query for readings is requested from a user, the Server communicates with the Historical Registry to obtain the requested information and returns it to the Web services. However, this approach improves the response time; it imposes energy consumption overhead due to continuously sending unnecessary data to the registry by sensor nodes.

## 3. The SOMM Model

In this section, first we present a short description of Service Oriented Architecture (SOA) and then we explain the main idea and the detail of the SOMM design.

### 3.1. SOA in a Nutshell

In the absence of a formal definition and reference model for SOA, it is difficult to classify architecture as service oriented. Although there has been some debate over the precise definition of SOA, there are a number of main concepts which have been agreed upon and which must be present in an implemented architecture such that it can be classified as an SOA. The most important of the main SOA concepts is that components should expose services in some way. These services are the basis upon which SOA is built and they allow for loosely coupled distributed computing using open standards-based protocols. Some type of central service repository is also required, in order for the different components to be able to advertise their own services and discover other services which they can use. The architecture should allow the different components to search for and discover services in the service repository. After discovering a service which a component may want to use, the component should be able to invoke the service with the required parameters and should obtain the resulting return value from the service. How these different concepts are implemented is not important. As long as they are present in the architecture, it can be classified as a SOA [24].

Service oriented architecture can be used as a suitable solution to control the complexities of development of WMSNs. Since SOA is a huge and complex concept and also because of limited resources available in a GWMSN, SOA must be customized before it can be used in a GWMSN. Therefore, here a lightweight prototype of SOA is used.

### 3.2. The Main Idea: Achieving a Dynamic Network Using SOA

Most early WMSN deployments have adopted *ad-hoc* and application-specific architectures. They were closed networks which typically were deployed by a single party (e.g., a government agency, a research institute or a private company) and there were no need to link them with each other. Only the owners of these networks used them and there were no other clients for them. In recent years, wide usages and different applications of WMSNs, the need for some kind of WMSNs which can perform multiple tasks and serve multiple clients at the same time raised. These networks are called GWMSNs (Generic WMSNs). They are expected to be open, multi-purpose, ubiquitous, and interoperable networks. A GWMSN can be replaced by multiple WMSNs. For example, consider two parties in a town which both of them deployed a WMSN throughout the town (e.g., traffic control organization and police station). Each network only can perform a special task for its owner and one party cannot use other one network. Also, if another party needs to monitor the town, it must deploy another WMSN. A GWMSN is able to replace these two networks and can service all parties at the same time. But, the design and development of a GWMSN using current design methods and programming frameworks, if it is possible, is a complex and costly task. Thus, new programming frameworks must be defined to enable the development of GWMSNs with reasonable cost.

**Figure 1.**
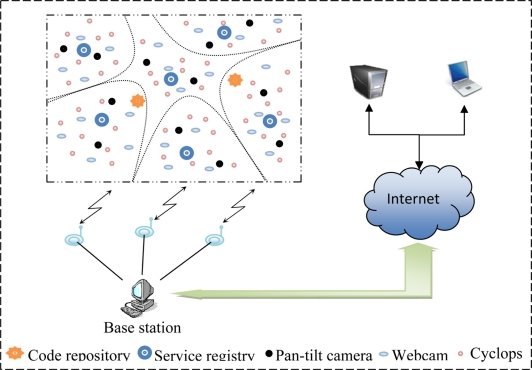
Overall view of a GWMSN designed based on SOMM.

The proposed middleware (SOMM) consists of some service registry and several servers. Servers register the specifications of their services, such as type and quality range of service, in the nearest service registry. A client can refer to appropriate service registry, find the address of server and communicate with server via message passing. Also, there are some special servers named code repository which are used as a place for storing different implementations of services that servers can provide to their clients. An overall view of a GWMSN which designed based on SOMM is depicted in Figure 1.

In this network, there are three types of video sensor nodes with different capabilities. Each video node registers specifications of its service in nearest service registry using API provided by SOMM. Clients can connect to base station via the Internet and query the SOMM for a specified service with a given QoS. After that, SOMM checks to see if it is possible for network to provide desired service with specified QoS. SOMM informs client if network cannot serve the client, otherwise, the SOMM connects the client to proper server node after reserving needed resources for that service. The details of these operations are described later.

It is clearly observable that using SOA concepts in SOMM lead to a fully dynamic and scalable network of server nodes which can provide different services to clients. A network can serve multiple clients at the same time and new functionalities can be added to the network easily (as described later).

### 3.3. Achieving Flexibility via Virtual Machine

As mentioned, due to the large number of nodes and unique constraints of a GWMSN, alteration is a natural characteristic of these networks. New nodes may be added to network or existing nodes may be removed. Also, because these networks are multi-purpose, new functionalities may be added or existing implementation of a service may be changed during lifetime of network. Current operating systems of WSNs support these kinds of changes poorly. To solve this problem, some virtual machines are developed for WSNs which have been described in Section 2. Employing a virtual machine as a middleware decrease the application code size; in addition, it simplifies the node reprogramming and as a result, increases the network flexibility. Portability is another advantage of virtual machine. A virtual machine lies as a layer between applications and the underlying operating system and makes them independent of operating systems. Having different implementations of virtual machine for various operating systems allows us to have multiple operating systems in a GWMSN at the same time. This is useful when using different operating systems for different nodes based on the node capabilities is needed. A virtual machine can also provide code mobility which is useful in application development for a GWMSN. Mobile agents allow a GWMSN to do multiple tasks and serve multiple clients at the same time. For these reasons, SOMM proposes a virtual machine with support for code mobility as its core.

#### 3.3.1. Virtual Machine and Code Mobility

A node in a GWMSN should be capable of providing multiple services. Hence, all nodes must have the code for all possible services that the GWMSN can provide. But, because of limitation in resources such as memory and processing capabilities in a GWMSN node, and also the fact that the size of multimedia applications are relatively large, it is not possible to have all the codes for all possible services in each node of GWMSN. Even if it is possible, the result is wasting valuable resources in the network. Also if there is a need to modify a service or add a new service to the network, the new code must be propagated to all nodes, which is a power consuming task and waste lots of energy.

SOMM proposes code mobility as a solution for the mentioned issues. The codes of different services are stored in a code repository (a rich node that has enough memory) as mobile agents. Server nodes download and run proper code for their services at runtime, considering requested QoS from their client. In this way, there is no need to store all codes in all nodes of network. Moreover, if a service changed or a new service should be added to the network, the new code should simply be sent to the code repository.

**Figure 2.**
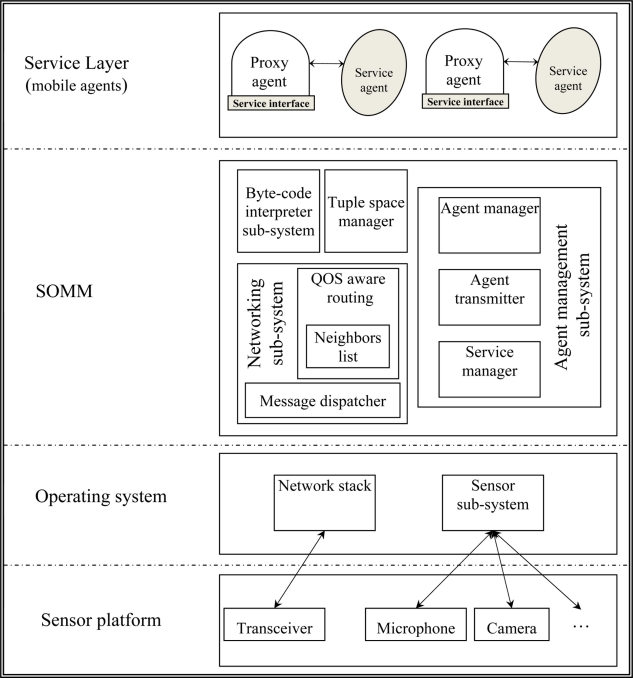
SOMM Architecture in a server node.

Figure 2 shows the SOMM architecture in a server node. Agent management sub-system is responsible for managing agent mobility. Each agent is an autonomous process which can migrate across nodes. An agent consists of instruction memory, data memory, program counter, operand stack and heap. During migration, the agent manager in source node, packs the agent and its execution context (which contains the necessary information for resuming agent execution in destination node) and then transfers the agent to the destination node using agent transmitter component. Agent transmitter component in destination node takes the transmitted agent and brings it to the agent manager. After that, agent manager unpacks the received agent and resume its execution.

Services in SOMM are provided by agents and there are two types of agents in SOMM:

- Proxy agent, that is responsible for providing a well defined interface for the corresponding service.
- Service agent, which encompasses the implementation of corresponding service.

A client connects to the proxy agent and declares its QoS requirements. The proxy agent queries the code repository and downloads and runs the suitable service agent for the corresponding service with the specified QoS requirements. The client does not have any information about the service agent and only communicates with the proxy agent. Communications between proxy and service agents also take place through tuple space. Using this approach, the implementation and interface of services are separated; modifying and replacing implementation of services can be done easily and also it is possible to have different implementations for a service considering different QoS requirements. For example, a server which provides multimedia streams to its clients can download and execute different encoding algorithms based on specified QoS by clients.

Byte-code interpreter sub-system is the core of SOMM and its main task is to interpret and execute the agents. Networking sub-system sits on top of operating system network stack and is responsible for initial configuration of network and also contains the needed routing protocols which we describe later.

#### 3.3.2. Routing and Network Configuration

**Figure 3.**
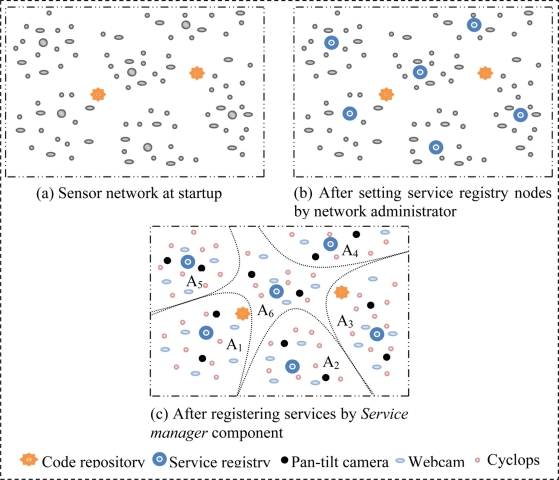
Network configuration phases at startup.

As depicted in Figure 3(a), at startup of a GWMSN which uses SOMM, the network consists of some code repository and some server node which do not provide any services. When a code repository starts (at network startup or when adding a new code repository to the network), it broadcasts a message named CR (Code Repository) to all of its neighbors which means ‘there is a code repository here’. CR message contains a numeric field called HC (Hop Count) that denotes the number of hops; the CR message should be broadcasted. When a node receives a CR message, after subtracting HC field by one, it stores the code repository address and broadcasts CR message to its neighbors if the HC field is non zero. Since all of the code repository nodes in SOMM are identical and all of them have all available agents, a node that receives two CR message only stores the address of code repository which has smaller HC value. This way, all nodes of network find closest code repository.

Service registries are also server nodes; this means that the operations of service registry nodes are done by agents. The network administrator specifies the service registry nodes at network startup and sends them the agent that can do service registry tasks. After that, this agent broadcasts a message called SR (Service registry) meaning that ‘there is a service registry here’ to all its neighbors. SR message like the CR message has a HC field. Then, all nodes of network find the closest service registry using a mechanism similar to that of finding code repository. Figure 3(b) shows the network after all nodes find closest code repository and service registry.

When all nodes find the address of the nearest code repository and service registry, the service manager component in each node identifies the available sensors and the type of service that each sensor can provide using API provided by underlying operating system. Subsequently, the service manager downloads the proper proxy agent for each service. All proxy agents register their services in the service registry and declare the QoS range which they can provide. At this time, network configuration is completed and services of nodes are accessible via closest service registry which also acts as cluster head as shown in Figure 3(c). In addition to automatically detecting the capabilities of node and downloading proper proxy agents, service manger is also responsible to provide the facilities for remotely deploying a service on the node. This means that the network administrator can remotely command the service manager to download and run a specified proxy agent form code repository.

Since routing is done using the nodes ID, service registry stores the server ID, QoS range and the availability of service in a table for each registered service. A base station allocates a unique name for each cluster head (which is a service registry node) and clients of network request their services based on these names. For example, in Figure 3(c), when the base station receives a request for a service of type X and quality of λ from cluster A_4_, it delivers the request to service registry A_4_. Service registry searches its table and sends the address of proper node which can provide aforesaid service to the base station. Then, the base station connects the client with the server node and the server node can serve the client.

Nodes in a GWMSN have very limited processing power and bandwidth; as a result, typically a node cannot service multiple clients at the same time. Therefore, a server node in SOMM before servicing a node sends a message named SCNA (Service Currently Not Available) to the service registry to state that it is currently busy and cannot serve other clients. When a service registry receives a SCNA message, it refers to its table and disables the corresponding service. After servicing the client, server node informs the service registry that it can serve new clients and then service registry again refers to its table and marks the corresponding service as available.

#### 3.3.3. Service Description

For a client to be able to interact with a service, the service must be well described. To this end, some standard languages like Web Service Description Language (WSDL) have been developed. WSDL is an XML-based language that provides a model for describing Web services. In SOMM, service registries also store service descriptions in WSDL format. However, nodes in SOMM do not support SOAP and HTTP bindings and only support TCP binding in binary format. This is because HTTP and SOAP bindings are text based and sending text messages causes more overhead compared to binary messages and increases the power consumption of nodes.

**Figure 4.**
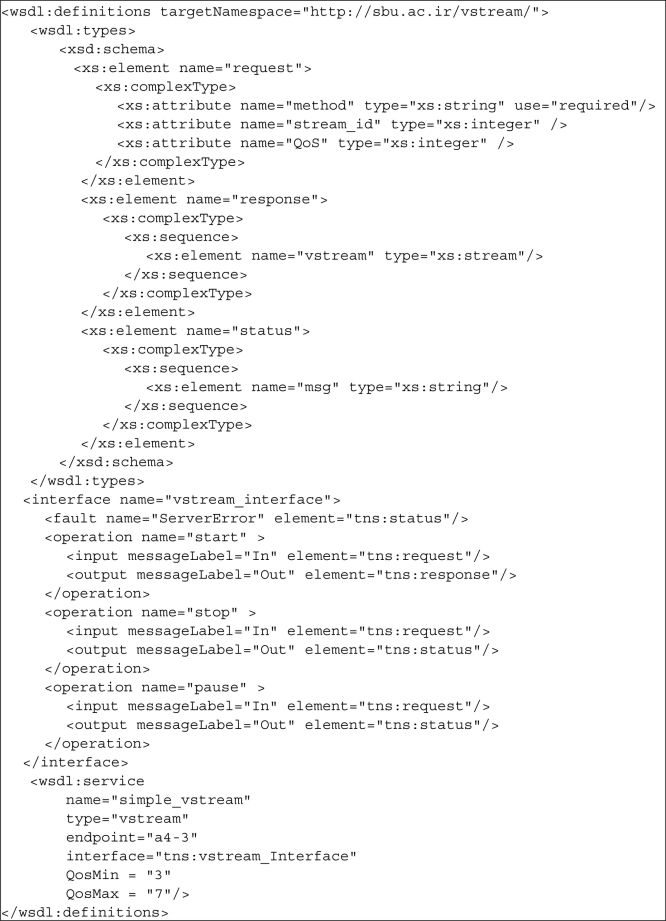
Service description for a simple video streaming service.

Each service in SOMM has four characteristics: service type, endpoint, QoS, and interface. As it is clear, the service type determines the type of service which is limited to the types of services that nodes of networks can provide. Endpoint defines the identifier of node that provides the service. QoS is the quality of service range that the provider node can provide and interface specifies the service operations and required parameters for each operation. Services in SOMM must be accessible from the outside of network; therefore, sink nodes which are the bridges between the network and the outside world, support SOAP and HTTP bindings. Users send their queries to sink nodes using SOAP or HTTP, then, the sink interprets the received queries, connects to the appropriate service registry and asks for the suitable services. Next, the sink extracts the address of server node and redirect the user requests to that node in binary format. Sink nodes act as the interpreters between the network users and network nodes. They convert the SOAP and HTTP messages to binary format and *vice versa*. In this way, services of network will be available over the Internet while controlling the power consumption of network. Figure 4 shows the service description for a simple video streaming service in SOMM. The service is hosted by node 3 of cluster a4 with the QoS range of 3–7 and as it is shown, its interface has three methods: start, stop and pause.

#### 3.3.4. Tuple Space

SOMM provides a tuple space on each node. A tuple space is a virtual repository or buffer that can contain tuples. The tuple space serves as an associative memory, in that tuples in the tuple space can be accessed by matching some or all the elements of the tuples to values or types presented in a template, which is simply a tuple set up for this matching.

**Figure 5.**
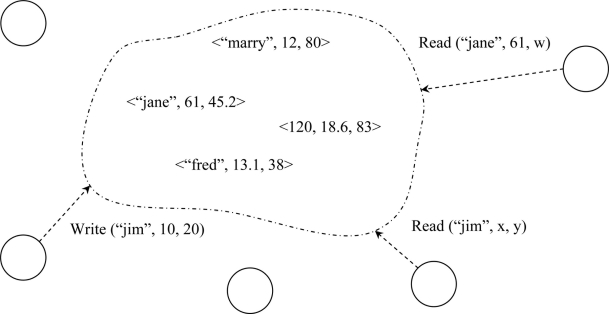
A symbolic tuple space. Read and write can be synchronous or asynchronous.

The concept of a tuple space was first described in 1982 in a programming language called Linda [25]. The basic idea is to have many active programs distributed over physically dispersed machines, unaware of others existence, and yet still able to communicate. They communicate to each other by releasing data (a tuple) into tuple space. Figure 5 shows a symbolic tuple space. Programs read, write, and take tuples from tuple space that are of interest to them. In general, a tuple captures the intuitive notion of an ordered list of elements like (120, 17, 8) or (“book”, “abcd”, “mo”, “pp”) or (“John”, 85, 15.7) which is accessed via pattern matching.

There are some other middlewares which proposed the using of tuple space in a WSN [26,27]. TinyLime [27] proposed a mechanism for communication between applications in different nodes of network using tuple space. In this approach, the tuple spaces of direct or indirect neighbor nodes are integrated and form a global tuple space. If an application in node A puts a tuple in its tuple space, applications in other nodes which share neighborhood with A can access that tuple. TeenyLime [26] also suggested a similar tuple space except that tuple spaces are only shared between nodes that are direct neighbors. However, since sharing tuple spaces between nodes of network imposes communication overhead, tuple spaces in SOMM are only accessible locally and agents can only access local tuple space. SOMM supports standard tuple space operations. An agent can add a tuple to local tuple space using an *out* command. The command *in* removes and returns a matched tuple from local tuple space. The *rd* command is similar to *in*, except that it does not remove the matched tuple from tuple space and only returns it to the caller agent.

#### 3.3.5. QoS Guarantee

An important aspect of designing a multimedia system is QoS guarantee. If a server accepts to provide a service to a client with a specified QoS, it must guarantee that the quality of service does not change as long as the service is not finished. Some algorithms are proposed to guarantee QoS in multimedia systems. RSVP [28] is one of these algorithms. The main idea in RSVP is to stabilize the QoS via reserving required resources. RSVP is a receiver-initiated QoS protocol. In other words, receivers are required to send reservation requests along the path to the sender. A sender in RSVP first sets up a path to potential receivers and provides the flow specification of the data stream to each intermediate node. When a receiver is ready to accept incoming data units, it first places a reservation request along its upstream path to the sender. When a sender receives a reservation request, it checks whether enough resources are available or not. The request is also passed to a policy control module to check whether the receiver has permission to make the reservation. If these two tests succeed, resources can be reserved.

In a GWMSN, reservation of resources in an intermediate node may be irrational; for the reason that nodes of networks have very limited energy, bandwidth and processing capabilities. Therefore, a node should not be an intermediate node of more than one stream. Consequently, in SOMM a simple protocol is proposed to lock the intermediate nodes of data streams, which is briefly described in the following paragraph.

When a client sends its request to the base station, the base station refers to the proper service registry and finds the address of server node. Then, it forwards the request of client to that server and also sends a reservation request to all intermediate nodes. All nodes of the network have a table which contains the information about their direct neighbors. This table also shows whether a neighbor is busy or not. When a node *n* receives a reservation request from the base station, it informs its neighbors that it is busy. Neighbor nodes then update the status of *n* in their table as a busy node and they won’t send their data through node *n*. Node *n* also informs the service registry that it is busy. After locking intermediate nodes, server can start sending data to client. When server completes its work, it notifies the intermediate nodes and service registry and they update their tables and mark the server node as a free node. Since wireless communications channel in a GWMSN is unreliable and also node failure is a prevalent event, locking mechanism may lead to a situation in which an intermediate node remains locked forever due to a message lost or server failure. To solve this problem in SOMM, each locked intermediate node of a stream uses a timer to keep the time since receiving last packet. If the timer exceeds a threshold, the locked node discerns that some problem happened to the stream and frees its resources. This threshold is a trade-off: assigning a small value to this threshold may lead to tearing a live stream and therefore losing the QoS guarantee. In another way, giving a large value to this threshold wastes the network resources. Determining the best value for this threshold in a general purpose framework may be a difficult task. But SOMM is a multimedia middleware and multimedia applications are generally real time applications and employ smooth streams. This smoothness in streams is a key for finding a good value for this threshold.

## 4. Application Programming Interface

The core of SOMM is a virtual machine which abstracts the complexities of underlying operating system API and facilitates the programming using a simple script language. The instructions which SOMM provides to programmer can be divided in four categories:

- **General purpose instructions:** This category includes instructions for mathematical operations, reading sensors, toggling LEDs, accessing heap and sending data. Also because SOMM is a stack-based virtual machine, it provides necessary instruction for manipulating stack. Some of these instructions are *add*, *halt*, *putled*, *rand*, *or*, *sense*, *eq*, *pop*, *pushc*, *pushacc*, *send*.
- **Migration instructions:** These instructions provide facilities for agents to migrate across nodes or copy themselves in another node. SOMM supports weak and strong migration. In weak migration, only the code of agent is transmitted to destination node and the migrated agent restarts its execution at destination node. But in a strong migration, in addition to code of agent, also its executing context is transmitted to destination node and as a result, migrated agent can continue its execution at destination node. The instructions in this category are *scopy*, *wcopy*, *smove* and *wmove* which stand for strong copy, weak copy, strong move and weak move respectively.
- **Service management instructions:** This group includes six instructions: *qsreg*, *qcrep*, *dlserv*, *regserv*, *disserv,* and *enserv*. The *qsreg* instruction allows the agent to query the service registry and search for a specified service. An agent can connect to code repository and gets the information about different implementations of a service using instruction *qcrep*. Instruction *dlserv* is for downloading a specified service from code repository. The *regserv* instruction is for registering a service in service registry and instructions *disserv* and *enserv* enables and disables a service in service registry, respectively.
- **Tuple space instructions:** This category contains the standard tuple space operations which an agent can manipulate local tuple space using them. These instructions are *out*, *in*, *inp*, *rd*, *rdp*. The instructions *in* and *rd* are blocking ones, this means that if they do not find any matching tuple, the calling agent blocks until a matching tuple is added to the tuple space. *rdp* and *inp* are non-blocking instructions and in the case that they do not find any matching tuple, the calling agent does not block.

**Figure 6.**
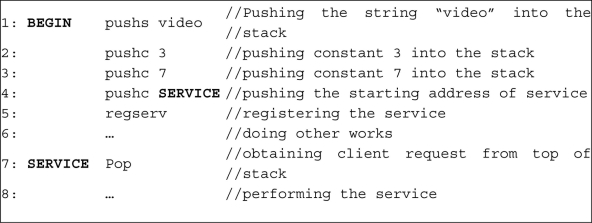
A sample code for registering a service in service registry using *regserv* instruction.

All of instructions pop their needed parameters from the top of the stack. It is the task of programmer to push the appropriate parameters into the stack in a proper order. For example, Figure 6 shows the sample code for using *regserv* instruction. In this piece of code, to register a video service with QoS range of 3–7, first the needed parameters are pushed to stack using instructions *pushc* and *pushs* and then the *regserv* is called for registering the service.

## 5. Implementation Details

While it is possible to implement SOMM on top of any operating system, current implementation of SOMM is based on TinyOS [14] that is the *de facto* standard operating system for WSNs. In the following sub-sections, we describe the key points of design and implementation of SOMM.

### 5.1. Agent Structure

The agent structure in SOMM is similar to that of Agilla [3], a middleware for WSNs with support for mobile agents which we described it briefly in Section 2. Each agent consists of stack, heap, and some registers. An agent can access the heap using *getvar* and *setvar* instructions. The heap and stack size is depended on the available memory in the node and can be altered when needed. Each agent also has three main registers: ID register for keeping agent ID, PC register for maintaining the address of next instruction, and accumulator register for storing the status of execution such as the result of comparing instructions.

### 5.2. The Key Interfaces

**Figure 7.**
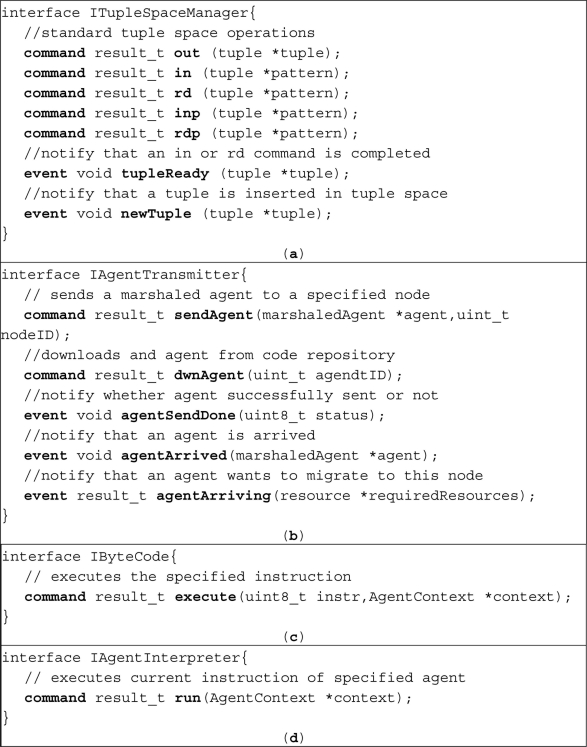
Key interfaces in SOMM (**a**) tuple space interface; (**b**) agent transmitter interface; (**c**) instruction interface; and (**d**) interpreter interface.

**Figure 8.**
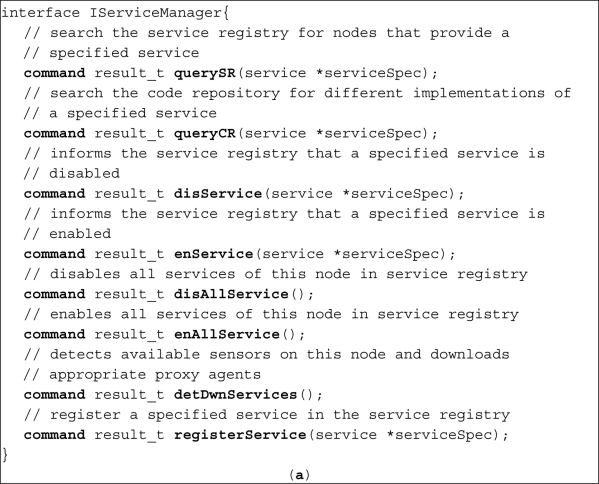

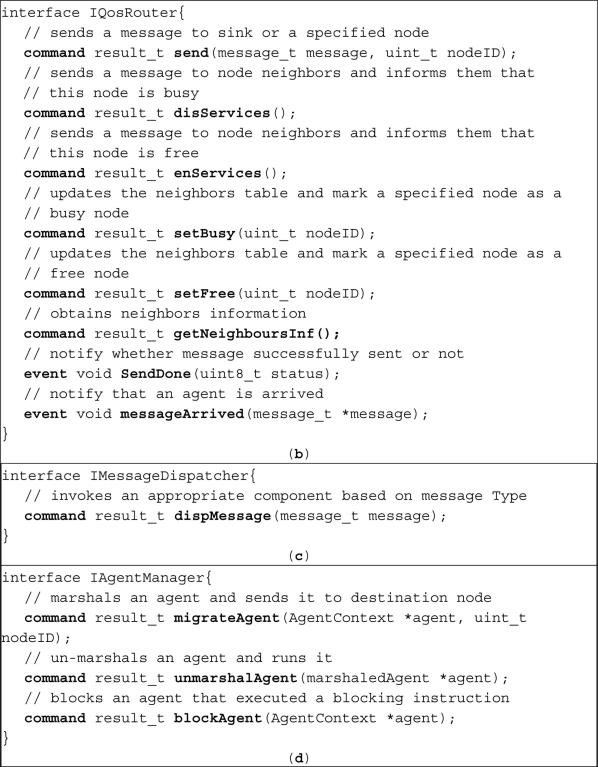
Key interfaces of SOMM (**a**) service manager interface; (**b**) QoS aware routing interface; (**c**) message dispatcher interface; and (**d**) agent manager interface.

Applications of TinyOS and also SOMM are written via a component based programming language called nesC [29]. Each component of a nesC program provides some interfaces. The key interfaces of SOMM are shown in Figures 7 and 8 while does not consists of much details for the purpose of readability.

Tuple space interface is shown in Figure 7(a) which in addition to provide standard tuple space operations, provides *tuple Ready* and *new Tuple* events. The *tuple Ready* event fires when an *in* or *rd* instruction is completed successfully and also *new Tuple* event fires when a new tuple is added to tuple space. Agent manager must be informed of addition of a new tuple to tuple space in order to resume the execution of a blocked agent that waits for that tuple and this is done using *new Tuple* event. The agent manager is also responsible for marshaling, un-marshaling, allocating memory and executing the agents. Therefore, it uses the *agent Arriving* event of agent transmitter component [that is shown in Figure 7(b)] to be aware when an agent wants to migrate to its host. Before an agent migrate to another node, the *agent Arriving* event of transmitter component in destination node fires and informs the agent manager. Agent manager then checks the available resources such as memory and decides whether to allow the migration or not. If migration is allowed, agent transmitter downloads the agent and informs the agent manager that the migration is completed using *agent Arrived* event. After that, agent manager un-marshals arrived agent and prepares it for execution.

Service manager interface which is shown in Figure 8(a) provides a command named *detDwnServices*. At node startup and after determination of closest code repository and service registry addresses, agent manager calls the *detDwnServices* command. As a result of executing this command, service manager detects the connected sensors to the node and downloads the proper proxy agent for each sensor.

### 5.3. The Key Components of Middleware and Their Relations

**Figure 9.**
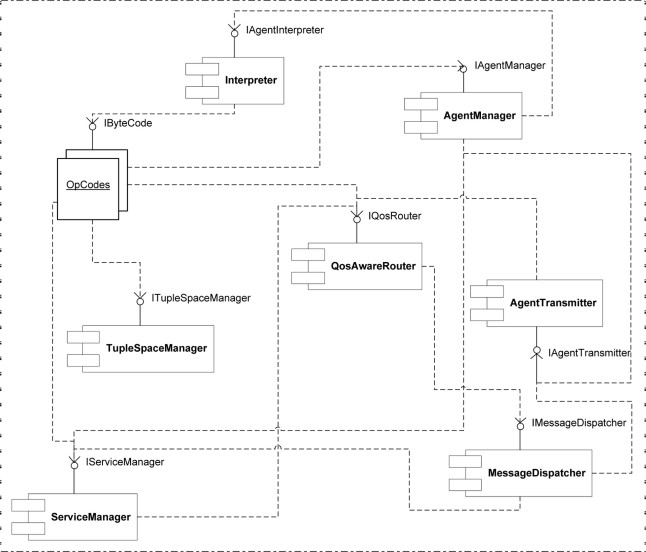
The key components of SOMM.

The key components of SOMM and their relationships are depicted in Figure 9. SOMM supports having multiple agents in the network and also in a node at the same time. However the locking mechanism provided by SOMM allows the programmer to prevent the node from providing multiple services at the same time, having multiple agents in a node is necessary. Because some situations may exist in which a node should provide multiple low quality services which do not need locking mechanism. Also since two types of agents exist in the SOMM (proxy agent and service agent), SOMM should provide facilities for simultaneous execution of these agents in a node.

When multiple agents exist in a node, it is important to share the CPU time between them. In SOMM, it is the agent manager component that performs this job. The agent manager executes agents using Round Robin policy. Scheduling in virtual machine based middlewares such as Agilla [3] and Mate [7] is at instruction level; which means that each time an executable entity (e.g., process, agent or capsule) grabs the CPU, the scheduler executes a fixed number of its instructions and then allocates the CPU to the next entity. For example, Agilla executes four instructions of an agent in each turn by default. SOMM also performs the similar way; which means that the agent manager that is responsible for executing agents, executes four instructions of the active agent in each turn.

As shown in Figure 9, agent manager uses the interpreter component to execute agents. To execute each instruction, agent manager calls the interpreter and the interpreter uses the corresponding Opcode component to execute the instruction.

**Figure 10.**
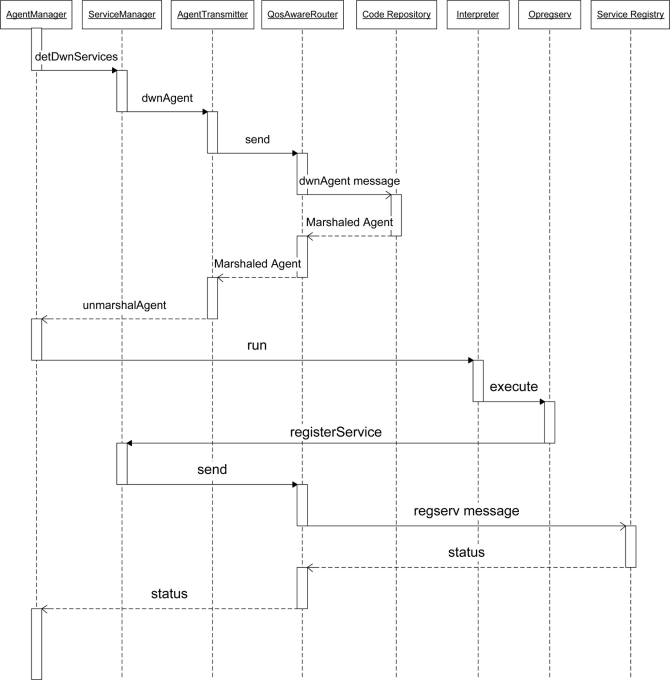
Sequence diagram for the startup process of a node.

To better understand the behavior of SOMM components, Figure 10 shows the sequence diagram for registering services scenario at the startup a node. As it is shown, agent manager calls the *detDwnServices* command of service manager; service manager detects the connected sensors using underlying operating system and service manager calls the *dwnAgent* command of agent transmitter to download the proxy agent for each sensor. Agent transmitter then downloads the appropriate proxy agent from code repository by sending and receiving messages to the code repository using QoS aware router component. When an agent transmitter downloads the agent, it delivers the agent to the agent manager and the agent manager executes the agent after un-marshaling it. Some of the details such as exchanged messages between agent transmitter and code repository during downloading agents are omitted from Figure 10 for the purpose of readability.

When a proxy agents starts, it registers its service in service registry using *regserv* command. To execute an agent, the agent manager calls the *run* command of interpreter and passes the agent as a parameter. When execution of a proxy agent reaches to the *regserv* instruction, the interpreter uses the Opregserv component to interpret the instruction. The Opregserv component is also calls the *registerService* command of the service manager. The service manager then registers the service by exchanging messages with the service registry using a QoS aware router and after that, the agent manager takes the control of CPU to execute another instruction.

## 6. Assessing Middleware Design

The objective of this section is to assess the effectiveness of SOMM in enabling a flexible, modifiable, reusable and scalable design of GWMSN applications. For this purpose, we discuss about how SOMM achieved its main goals and after comparing SOMM with other middlewares, we propose a case study to show the usefulness of SOMM.

### 6.1. Middleware Design Goals

As we mentioned earlier, the main goal of SOMM is enabling low cost design and development of modifiable and scalable applications for GWMSNs. In the following, we discuss about the question that whether SOMM has reached its goals or not:

#### 6.1.1. Modifiability

Whenever the cost of modifying a system after its deployment is smaller, it is said that the system is more modifiable. As the lifetime of a system become longer, the probability that the system need to be modified increases and the modifiability of the system becomes more important. Due to the long lifetime of WSNs, the modifiability of WSN application has high importance, but current programming frameworks for WSNs does not provide satisfactory facilities for developing modifiable applications and therefore the cost of modifying a WSN application is very high. For example if an application needs to be changed, all nodes of network must be reprogrammed. Reprogramming all nodes of the network consumes a lot of energy and hence, it is a costly task. To increase modifiability, SOMM uses a virtual machine which supports mobile agents. Virtual machine makes the applications much smaller and as a result, decreases the cost of modifying them. Having small applications also decreases the energy consumption and cost of reprogramming. SOMM also defines some code repository in the network as places for storing mobile agents. These two concepts highly increase the modifiability of the applications. Applications can be defined as multiple agents collaborating with each other and modifying and replacing an agent is as easy as only replacing it in code repository and there is no need to reprogram all node of network.

#### 6.1.2. Scalability

In a WSN, scalability can be seen from two points of view. From the first, scalability stands for software scalability and from the second, scalability refers to hardware scalability. Software scalability means that extending network applications and adding new functionalities can be done easily. Also, hardware scalability refers to the ease of adding new nodes with different capabilities to the network. SOMM improves both hardware and software scalability. Using SOMM, adding new nodes to a network is as easy as installing the SOMM on nodes and adding them to the network. SOMM automatically detects the hardware capabilities of new nodes and downloads appropriate services for them. In the absence of SOMM, all nodes must have all applications and therefore to add new program to the network, it must be sent to all nodes. Consequently, the number of applications in a network is bounded by the limited memory of nodes and this limits the software scalability. SOMM solves these problems by using code repositories and service registries. In the presence of SOMM, services are kept in code repositories and therefore the maximum number of services in SOMM is bounded to the available memory in code repository nodes which are rich nodes regarding to available memory. Nodes can download codes of services based on their needs and service registry nodes help the clients to find the appropriate node which can provide their needed services.

### 6.2. Comparing with Other Middlewares

In this section, we compared SOMM with previously mentioned middlewares by concentrating on how well they meet the design criteria. Table 1 shows the main features of these middlewares beside the level that each middleware satisfies each of design criterions and in the follow, these features have been described in more detail.

#### 6.2.1. Scalability

Mate supports scalability by using active messages [30] to update the network protocols and parameters by injecting new capsules. It facilitates the addition of new nodes to the network but the number of concurrent applications in Mate is very limited because of maintaining all applications in all nodes of network. Cougar and TinyDB use a centralized optimizer to maintain a global knowledge of the network and since the dynamic nature of large-scale sensor networks poses a problem for this centralized approach, these middlewares do not support scalability. MiLAN tackles the scalability challenge by providing application driven network management. Agilla and Impala provide application adaptation at runtime and therefore scalability using mobile agents and a suitable architecture model. Kairos is not scalable because it does not provide the ability for applications to fully control the underlying runtime resources. TinySOA provides a highly scalable framework by using service oriented architecture. Also as we mentioned in previous sub-section, SOMM provides highly scalable platform by using SOA and the concepts of code repositories and service registries and also the ability to automatically detect and register capabilities of nodes.

#### 6.2.2. Power Awareness

Mate and Agilla are not efficient in term of power awareness. Their virtual machine and instruction interpreter increases the energy consumption of network. However, Agilla provides an energy efficient mechanism for application modification by using mobile agents that can be updated separately, but if an agent in Agilla needs to be replaced with a new one, the new agent should be propagated to all nodes of network and this task consumes a lot of energy. Impala supports energy efficient application modification by allowing the application to be as modular as possible. The key idea is changing a small module of an application needs less energy with comparison to updating the whole application. MiLAN supports energy efficiency by dynamically configuring the underlying network protocol based on the specified QoS by the application. This middleware tunes the network parameters at runtime regarding to application needs and with the goal of minimizing the energy consumption of network. Cougar is not energy efficient because it uses the worthful resources to transfer the large amounts of unprocessed data from sensor nodes to its database server. Mate supports the energy efficiency by providing grouped aggregation queries using aggregation function. This results in an appreciable bandwidth and energy savings by decreasing the amount of data that should be transferred through the network. Also TinySOA is not fully power aware because it transfers and caches all sensor reading to its database which wastes the resources of network. However SOMM increases the energy efficiency of application modification and node reprogramming by using the concepts of mobile agents and code repositories, the virtual machine used in SOMM introduces some overhead due to its instruction interpretation.

#### 6.2.3. Modifiability

Agilla and Mate support modifiability by using virtual machines. Thanks to the virtual machine approach, applications in Mate and also agents in Agilla can be updated without need for reprogramming sensor nodes. Also, it is possible to add new applications to the network after deployment. Cougar and TinyDB are modifiable since adding new capabilities requires modifying the query processor in all sensor nodes. MiLAN is also not modifiable because of its tight coupling with applications. Impala supports modifiability by using mobile agents and modular programming. A module of an application can be updated without need to update the whole application. As mentioned in the previous sub-section, modifiability in SOMM is supported via mobile agents and code repositories.

#### 6.2.4. Heterogeneity

Mate and Agilla provide APIs supplemented by virtual machines and hence they support heterogeneity. Impala is designed to run only on Hewlett-Packard/Compaq iPAQ Pocket PC handhelds running Linux and it does not support heterogeneity of sensor nodes. Cougar and TinyDB which try to manage large scale networks in a centralized fashion do not support heterogeneity, since heterogeneity of nodes in a large scale sensor network increases the complexities of centralized management of them. MiLAN is an application driven middleware and the tight coupling between this middleware and its applications results in the lack of support for operating systems and hardware heterogeneity. TinySOA is developed on top of TinyOS operating system and utilizes the platform heterogeneity provided by this operating system and hence it can be installed on different hardware platforms that TinyOS supports. SOMM is capable of handling heterogeneous nodes with different capabilities and also because of its use of virtual machines; it is possible to have different platforms with different operating systems in the network if needed.

#### 6.2.5. Ease of Use

The only mechanism which Mate and Agilla provide for developing applications is byte code programming. Thus, these middlewares are not very easy to use. A high level language is needed to help programmers with the task of programming. Impala makes the task of programming easy by providing a modular programming API.

Cougar and TinyDB offer an easy-to-use database query system for different network operations. These middlewares provide an abstract view of the network to the user as a single entity and make the distribution issues hidden. As a result, the user does not need to deal with low-level APIs in the sensor node. MiLAN helps the programmer with an easy to use approach which is concentrated on high-level abstractions. TinySOA provides a set of web services which programmers can write applications to employ them. This way, programmers can easily obtain network services using any programming language that supports web service communication. Since current implementation of SOMM only comprises byte code, it is not very easy to use; a high level language and programming model for application development are needed in order to simplify the programmer’s task.

#### 6.2.6. General Purpose

All the mentioned middlewares are general purpose middlewares, excluding Impala which is specially designed for a wildlife tracking project called ZebraNet. SOMM is a special purpose middleware designed to support multimedia transmission in a sensor network. However, it is possible to use this middleware in other types of WSNs.

### 6.3. Usability Case Study

In this section, we propose a case study to assay the capabilities of SOMM for developing applications for a real world scenario.

Consider a sensor network like the one in Figure 1 spread throughout a city. Suppose that the nodes of this network are multimedia nodes with different capabilities. The traffic control organization (TCO) and the police are two clients of this network. The TCO continuously uses the network and collects the traffic information of whole city, but the police use the network only when they need to monitor an area of the city. When the police uses the network, it needs a high quality service and is also takes priority over TCO. We want to use SOMM to develop necessary applications for this scenario.

The TCO application which runs continuously must be very energy efficient. Therefore, it provides two types of services. The first service is a low quality service and instead of sending a video stream for its client, it periodically extracts important information such as average speed and number of cars and sends them to its client. It can detect unusual events like accidents and also can search its field of view for a specified car and inform the client. The second service is a video streaming service which streams video to client considering its QoS requirements. Normally, the TCO uses the first service in order to save network’s resources. If received information shows an abnormal event like dense traffic or an accident, the TCO employs the second service to precisely monitor that event and make proper decisions.

The police needs a tracking service to carry out their missions. This service also should preserve the QoSto where it is possible. Suppose that a car is stolen and the police suspect that the stolen car is in a wide area of the city and therefore it should monitor the whole area to find the car. The police sends the characteristics of stolen car to all the nodes in that area and asks them to announce when they detect the car. When a node detects the stolen car, it informs the police and at this time, the police makes use of the tracking service. The agent that is responsible for tracking service, as well as sending a video stream to its client also detects the direction of the target and finds the node that the target is going toward its field of view and clones itself on that node. Using this technique, the tracking agent can precisely track its target.

Suppose that the target of tracking agent arrives to an area that is under monitoring of TCO using its second service. As we mentioned, the TCO’s second service is a high quality service and it locks the intermediate nodes in order to preserve its QoS. The tracking agent needs to clone itself to a node that is locked by the TCO service. Although SOMM does not provide any facilities for priority based resource management in the network, it is possible to solve the contention problem between the TCO service and the police tracking service using SOMM primitives. To solve this problem, all we need is an agreement on a predefined tuple template between the TCO service and tracking service. This tuple template is for notifying the TCO service that the police needs its node. When the tracking agent reaches a node that is under control of the TCO service, it puts a tuple with the predefined template in tuple space of that node. When the TCO service detects the mentioned tuple, it informs its client that the node is under control of the police and stops sending data. After the tracking agent finished its work and released that node, the TCO service on that node can resume its work.

## 7. Conclusions and Future Works

This paper has presented the so-called SOMM middleware, a service oriented middleware which is developed to support the programmers of GWMSNs applications. The main goal of SOMM was to enable the development of modifiable and scalable applications for GWMSNs. SOMM uses mobile agents as the entities which provide services to network clients and also the concept of code repository as a place to store different agents. In a network which uses SOMM, multiple agents are executed at the same time. This way the network can handle multiple clients simultaneously. Although our initial investigations clearly demonstrate the usefulness of SOMM to support a wide range of GWMSN applications, we also plan to extensively evaluate both qualitatively and quantitatively the advantages brought by SOMM to the application developers, e.g., in terms of code complexity and inter-service dependencies. Also for the reason that the programming using byte-code is a tedious task, we plan to develop a high level language for SOMM to further simplify the task of programming for GWMSNs.

## Figures and Tables

**Table 1.** Comparing SOMM with other middlewares.

**Middleware**	**Key features**	**Scalability**	**Power awareness**	**Modifiability**	**Heterogeneity**	**Ease of use**	**General purpose**
Mate [7,8]	Virtual machine, small size applications, permodule reprogramming	Medium	Medium	Medium	Medium	Low	High
Cougar [12]	Virtual relational database, Abstract DataTypes, SQL Like Language, In-network query processing	Low	Medium	Low	Low	High	High
MiLAN [11]	Macro-programming with high level concerns, QoS based efficient execution planning, Network protocol stack	Low	High	Low	Low	High	High
Middleware	Key features	Scalability	Power awareness	Modifiability	Heterogeneity	Ease of use	General purpose
TinyDB [4]	Virtual relational database, Acquisitional query processing, Semantic routing tree, Power aware query optimization	Low	High	Low	Low	High	High
Agilla [3,10]	Virtual machine, Autonomous mobile agents, Tuple space, supporting self-adaptive applications	Medium	Medium	Medium	Medium	Medium	High
Kairos [13]	Macro-programming, Caching with eventual consistency, Language independent	Low	Medium	Medium	Low	High	High
Impala [9]	Modular programming, Mobile agents, Dynamic code update	Medium	Medium	Medium	Low	Medium	Low
TinySOA [22,23]	Service oriented model, Internet accessible via web services	High	Medium	Medium	Medium	High	High
SOMM	Multimedia support, QoS awareness, Service oriented model, Virtual machine, Mobile Agents, Tuple space	High	Medium	High	High	Medium	Medium
